# Clinical and economic burden associated with cardiovascular events among patients with hyperlipidemia: a retrospective cohort study

**DOI:** 10.1186/s12872-016-0190-x

**Published:** 2016-01-14

**Authors:** Kathleen M. Fox, Li Wang, Shravanthi R. Gandra, Ruben G. W. Quek, Lu Li, Onur Baser

**Affiliations:** Strategic Healthcare Solutions, LLC, Monkton, MD USA; STATinMED Research, Plano, TX USA; Amgen Inc, Thousand Oaks, CA USA; Center for Innovation & Outcomes Research, Department of Surgery, Columbia University, New York, NY USA; MEF University, Istanbul, Turkey; Strategic Healthcare Solutions, LLC, 133 Cottonwood Creek Lane, Aiken, SC 29803 USA

**Keywords:** Hyperlipidemia, Cardiovascular events, Clinical burden, Economic burden

## Abstract

**Background:**

Annual direct costs for cardiovascular (CV) diseases in the United States are approximately $195.6 billion, with many high-risk patients remaining at risk for major cardiovascular events (CVE). This study evaluated the direct clinical and economic burden associated with new CVE up to 3 years post-event among patients with hyperlipidemia.

**Methods:**

Hyperlipidemic patients with a primary inpatient claim for new CVE (myocardial infarction, unstable angina, ischemic stroke, transient ischemic attack, coronary artery bypass graft, percutaneous coronary intervention and heart failure) were identified using IMS LifeLink PharMetrics Plus data from January 1, 2006 through June 30, 2012. Patients were stratified by CV risk into history of CVE, modified coronary heart disease risk equivalent, moderate- and low-risk cohorts. Of the eligible patients, propensity score matched 243,640 patients with or without new CVE were included to compare healthcare resource utilization and direct costs ranging from the acute (1-month) phase through 3 years post-CVE date (follow-up period).

**Results:**

Myocardial infarction was the most common CVE in all the risk cohorts. During the acute phase, among patients with new CVE, the average incremental inpatient length of stay and incremental costs ranged from 4.4–6.2 days and $25,666–$30,321, respectively. Acute-phase incremental costs accounted for 61–75 % of first-year costs, but incremental costs also remained high during years 2 and 3 post-CVE.

**Conclusions:**

Among hyperlipidemic patients with new CVE, healthcare utilization and costs incurred were significantly higher than for those without CVE during the acute phase, and remained higher up to 3 years post-event, across all risk cohorts.

## Background

The global cost of cardiovascular disease (CVD) is estimated at $ 863 billion and is estimated to rise to $ 1,044 billion in 2030 [1]. The American Heart Association has estimated the direct costs for CVD in the United States at $195.6 billion, approximately 61 % of the total CVD-related healthcare costs [2]. Additionally, hyperlipidemia was among the top 10 costliest medical conditions in 2008 in the US adult population [3]. Presence of hyperlipidemia directly correlates with the risk of developing coronary heart disease (CHD) and future cardiovascular (CV) events [4]. Less than half of adults with elevated low density lipoprotein cholesterol (LDL-C) levels receive treatment or are adequately treated [5, 6] and as a result, high-risk patients continue to remain at risk for new CV events. Almost 44 % of the US population is projected to be diagnosed with some form of CVD by 2030 [2]. These factors result in a substantial clinical and economic burden in terms of direct healthcare utilization and costs.

While several studies have examined the economic burden of CV events [7–12], to our knowledge contemporary and long-term analyses concerning these event costs incurred by hyperlipidemic patients across a range of CVD risk levels is not available. Previous studies focused on short-term healthcare costs due to CV events [13–17] and investigated patient populations diagnosed with acute coronary syndrome [13, 14], hypertension [15], atherosclerosis [16] or diabetes [17], but not hyperlipidemia. Furthermore, prior studies focused only on the initial CV event and therefore, limited data are available regarding recurrent and subsequent CV event costs. Prior studies have investigated the economic burden of CV events over various time periods [10]; however, incremental costs among hyperlipidemic patients with and without CV events, and in particular, costs stratified by CVD risk level and associated with myocardial infarction (MI), ischemic stroke (IS) unstable angina (UA), coronary artery bypass graft (CABG), percutaneous coronary intervention (PCI), heart failure (HF) and transient ischemic attack (TIA), all in one study, have not been previously examined. Therefore, the present study is one of the first to estimate the short-term and long-term (up to 3 years) direct clinical and economic burden of new CV events among hyperlipidemic patients at different CVD risk levels and by specific CV event type.

## Methods

### Study design

We conducted a retrospective cohort study including patients with a hyperlipidemia diagnosis who had a new CV event matched to patients without new CV events, using the IMS LifeLink PharMetrics Plus dataset for the study period January 1, 2006 through June 30, 2012. This nationally-representative longitudinal database contains medical and pharmacy claims for over 50 million commercially-insured patients throughout the United States [7, 18, 19]. All claims data were from a limited dataset with de-identified patient information. No patients were directly involved in the study; therefore, this study was exempt from an Institutional Review Board review.

### Study population

Patients (age ≥18) were included in the study if they had ≥1 medical claims for hyperlipidemia (International Classification of Diseases, 9th Revision Clinical Modifications [ICD-9-CM] code 272) [20] from January 1, 2006 through June 30, 2009. The first diagnosis claim date was designated as the hyperlipidemia diagnosis date. As detailed in Appendix 1, patients were required to have at least one inpatient medical claim for a new CV event (MI, IS, UA, TIA, HF, CABG and PCI) after the hyperlipidemia diagnosis date and during the identification period (January 1, 2007 through June 30, 2009). For hyperlipidemic patients with a new CV event, the earliest inpatient claim date was designated as the index date. If a patient had more than one inpatient claim for a new CV event on the index date, only one CV event was selected, according to the following hierarchy: MI, IS, UA, HF, TIA, CABG and then PCI, based on the clinical importance (e.g. acute/urgency) of CV events and CV-related procedures. The comparison group included patients with no new CV event after the hyperlipidemia diagnosis and through the end of the study period (June 30, 2012). Baseline period of the 12 months prior to the index date was utilized to characterize patients’ CV risk level (e.g. history of CVE or diabetes) and comorbidity status. Patients were followed from the index date through 3 years post-index date to estimate short-term (first 30 days and 1 year) and longer-term (2 years and 3 years) direct costs.

Matching was completed in a two-step approach. The first step was 1:1 match (age, gender, US region) to assign an index date for patients without a new CV event and define the baseline period for quantifying baseline characteristics (CV risk level, comorbidities). These initially matched patients with no new CV event were then assigned the same index date as that of their matched patients who had a new CV event. This assignment of index date to patients with no new CV event provided the same baseline and follow-up time periods for the comparison of outcomes between patients with and without new CV events.

The second step of matching, propensity score matching (PSM) with 0.01 calipers, was applied to control the differences in baseline clinical and demographic factors between patients with and without new CV events within each risk cohort [21, 22]. A standardized difference (STD) of >10 % was used to assess significant practical differences in the case–control comparison [23]. The baseline variables adjusted in the model were age group, gender, US region, Charlson comorbidity index (CCI) score, Chronic Disease Score (CDS), individual comorbid conditions (hypertension, arrhythmias, metabolic syndrome, liver disease, obesity, and chronic kidney disease) and number of inpatient admissions per patient per month (PPPM). The methods used in this study have also been published in prior literature [7, 10]. The CCI score is based on ICD-9 codes and CDS uses pharmacy dispensation information for specific drug classes to estimate the burden of comorbidities [24]. The CCI and CDS score have been widely used in many retrospective studies [25–28].

**Fig. 1:**
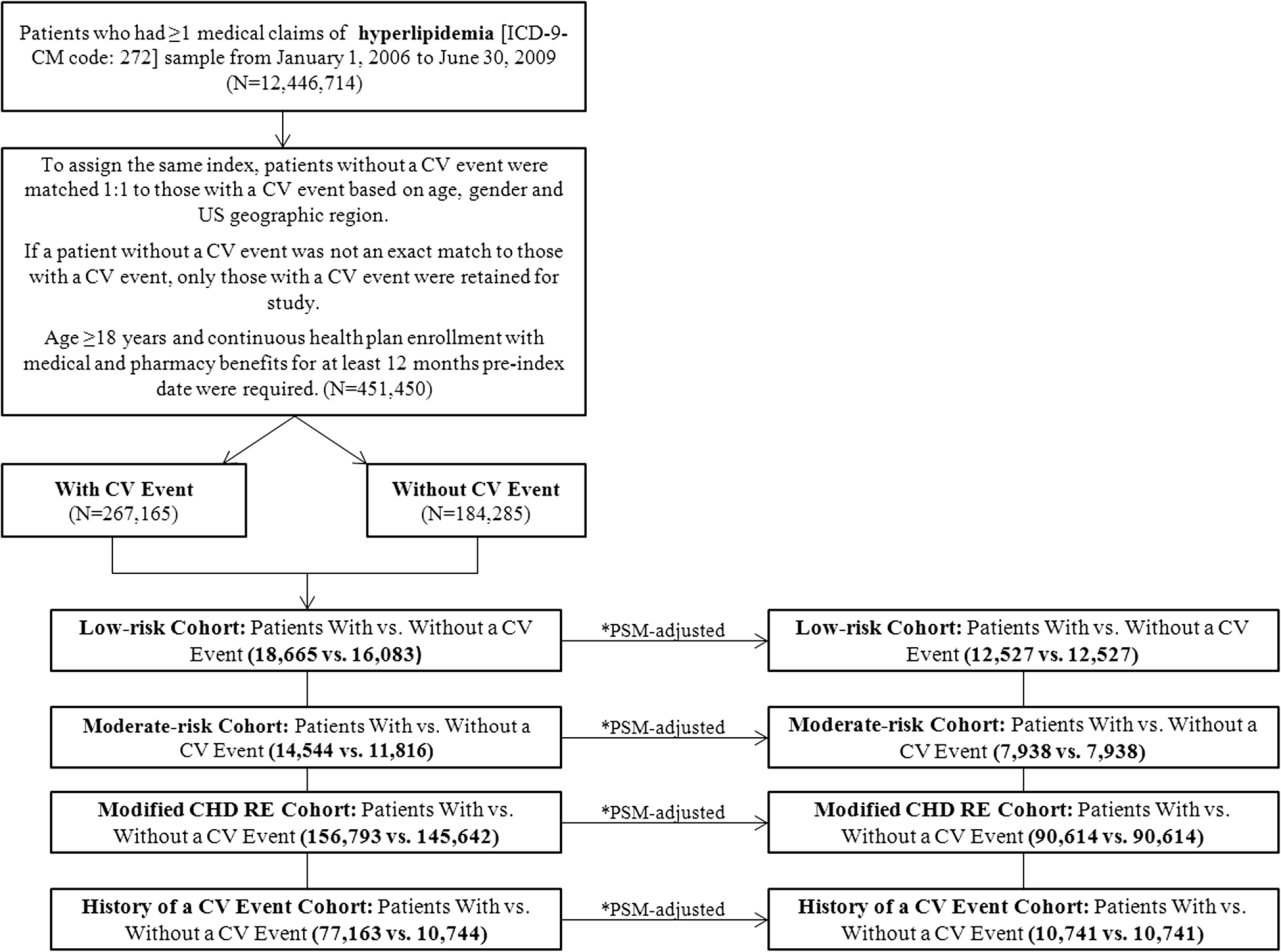
Patient Selection Flowchart. *Propensity score matching was applied for each cardiovascular disease risk cohort using covariates: age group, gender, US region, baseline Charlson comorbidity index score, Chronic Disease Score, comorbidities and number of inpatient admissions per patient per month. CV: cardiovascular; CHD RE: coronary heart disease risk equivalent; PSM: propensity score matching

Based on the risk level during the 12-month pre-index (baseline period), the study sample was subdivided into the following CVD risk cohorts: history of CV event, modified CHD risk equivalent (RE), moderate risk and low risk (Fig. 1). Risk levels were defined based on the National Cholesterol Education Program (NCEP) Adult Treatment Panel (ATP) III guidelines [29] (Appendix 2).

The history of CV event cohort included patients with MI, UA, CABG, PCI or IS, the modified CHD RE cohort included patients with peripheral arterial disease, abdominal aortic aneurysm, coronary artery disease, diabetes or dyslipidemia. Patients with at least two of the following three risk factors a) hypertension and/or pharmacy claim for blood pressure-lowering medication, b) aged ≥45 for men and aged ≥55 for women, c) pre-index high-density lipoprotein cholesterol <40 mg/dl were included in the moderate risk cohort, and patients with zero or one risk factor were included in the low risk cohort [see Appendix 2 for detailed ICD-9-CM codes]. Outcome measures included distribution of CV event type, healthcare utilization, and direct incremental costs (obtained from claims) incurred during the acute (first 1 month post-index date), and long-term (1, 2, 3 years post-index date) follow-up periods for hyperlipidemia patients, stratified by CVD risk level. Healthcare utilization included inpatient, outpatient, outpatient office, emergency room and pharmacy visits and direct costs associated with healthcare utilization were computed from health plan- and patient-paid amounts. Total costs included inpatient, outpatient and pharmacy costs. Costs were adjusted to 2012 US dollars using the annual medical care component of the consumer price index (CPI) to reflect inflation.

### Statistical analysis

#### Descriptive analysis

Descriptive analysis was conducted to compare demographic and clinical characteristics between patients with and without a new CV event within each risk cohort. The direct total incremental costs were calculated as the difference in total costs for patients with a new CV event and total costs for patients without a CV event. Negative incremental costs indicate that the total costs were lower for patients with new CV events than for patients without new CV events.

#### Multivariate analysis

The differences in economic outcomes for each risk cohort were compared among PSM cases and controls. Patients without new CV events were designated as the reference group (controls). All analyses were performed using SAS® version 9.3 (SAS® Institute Inc., Cary, NC).

## Results

Among patients with a new CV event, a large proportion had two or more new CV events (65.8 %) during the 3-year follow-up period. Second and subsequent CV events during follow-up were often the same event type as the first event. A total of 451,450 patients were eligible for the study, among which 267,165 patients had a new CV event, and 184,285 patients had no new CV event before 1:1 matching. A total of 184,285 hyperlipidemic patients with new CV events from January 1, 2006 through June 30, 2009 were matched according to age, gender and US region to 184,285 hyperlipidemic patients without a new CV event (Fig. 1).

### Baseline demographic and clinical characteristics

Baseline demographic and characteristics of unmatched patients with a new CV event (*N* = 267,165) and patients without a new CV event (*N* = 184,285) are provided in Appendix 3. Baseline demographic and clinical characteristics for propensity score-matched patients with a new CV event (*N* = 121,820) and patients without a new CV event (*N* = 121,820), stratified by CVD risk level, are provided in Table 1. Patients without CV events were well-matched with patients with new CV event within each risk cohort, since the STD was <10 % for all variables included in the PSM. The majority of patients were classified in the modified CHD RE cohort (74.4 %), followed by the history of CV event cohort (8.8 %). Overall, the average age of patients with a new CV event (*N* = 121,820) ranged from 56 to 72 years; 65–67 % were male; and hypertension was the most common baseline comorbidity (4.7–84.4 %).

**Table 1:** PSM-adjusted 12-month pre-index demographic and clinical characteristics for hyperlipidemic patients with and without new CV events

	History of CV event cohort				Modified CHD RE Cohort				Moderate risk cohort				Low risk cohort			
	Without CV events	With CV events			Without CV events	With CV events			Without CV events	With CV events			Without CV events	With CV events		
	(*N* = 10741)	(*N* = 10741)			(*N* = 90614)	(*N* = 90614)			(*N* = 7938)	(*N* = 7938)			(*N* = 12527)	(*N* = 12527)		
	Mean [%]/(SD)	Mean [%]/(SD)	P-value^a^	STD	Mean [%]/(SD)	Mean [%]/(SD)	P-Value^a^	STD	Mean [%]/(SD)	Mean [%]/(SD)	P-value^a^	STD	Mean [%]/(SD)	Mean [%]/(SD)	P-value^a^	STD
Age	73.66(13.15)	71.76(12.18)	<0.0001		65.32(12.95)	64.69(12.75)	<0.0001		65.58(11.93)	65.45(12.11)	0.503		56.18(11.24)	55.86(10.92)	0.022	
18–24	[0.0 %]	[0.0 %]	N/A	0.0	[0.0 %]	[0.0 %]	0.336	0.5	[0.0 %]	[0.0 %]	N/A	0.0	[0.1 %]	[0.1 %]	0.683	0.5
25–34	[0.1 %]	[0.1 %]	0.781	0.4	[0.3 %]	[0.3 %]	0.189	0.6	[0.0 %]	[0.0 %]	N/A	0.0	[1.1 %]	[1.1 %]	0.952	0.1
35–54	[7.0 %]	[6.0 %]	0.006	3.8	[19.0 %]	[19.7 %]	<0.0001	1.9	[14.6 %]	[14.5 %]	0.946	0.1	[45.6 %]	[45.6 %]	0.970	0.1
55–64	[22.1 %]	[20.9 %]	0.043	2.8	[36.7 %]	[36.4 %]	0.1	0.8	[43.9 %]	[43.8 %]	0.949	0.1	[36.9 %]	[36.9 %]	0.990	0.0
≥65	[70.9 %]	[73.0 %]	0.001	4.6	[44.0 %]	[43.6 %]	0.129	0.7	[41.6 %]	[41.7 %]	0.910	0.2	[16.3 %]	[16.3 %]	0.932	0.1
Male	[66.6 %]	[65.2 %]	0.029	3.0	[65.3 %]	[65.2 %]	0.657	0.2	[66.7 %]	[66.7 %]	0.946	0.1	[64.7 %]	[64.7 %]	0.926	0.1
US geographic region																
Northeast	[39.3 %]	[39.5 %]	0.769	0.4	[35.5 %]	[35.3 %]	0.366	0.4	[32.1 %]	[32.1 %]	0.973	0.1	[35.2 %]	[35.3 %]	0.874	0.2
Midwest	[22.1 %]	[23.3 %]	0.040	2.8	[25.9 %]	[26.0 %]	0.351	0.4	[28.2 %]	[28.3 %]	0.930	0.1	[26.5 %]	[26.5 %]	0.989	0.0
South	[24.4 %]	[22.6 %]	0.002	4.3	[27.6 %]	[27.6 %]	0.992	0.0	[28.3 %]	[28.1 %]	0.874	0.3	[28.9 %]	[28.8 %]	0.933	0.1
West	[14.2 %]	[14.7 %]	0.351	1.3	[11.1 %]	[11.1 %]	0.929	0.0	[11.4 %]	[11.5 %]	0.960	0.1	[9.4 %]	[9.3 %]	0.914	0.1
Baseline comorbid condition																
CCI Score	2.72(2.15)	2.82(2.14)	<0.001	4.9	1.23(1.54)	1.21(1.47)	<0.001	1.7	0.32(0.77)	0.31(0.75)	0.876	0.3	0.14(0.5)	0.14(0.49)	0.848	0.2
Chronic disease score	5.29(4.06)	5.49(4.19)	<0.001	5.0	4.58(3.62)	4.57(3.65)	0.716	0.2	3.99(2.86)	3.99(2.87)	0.892	0.2	0.97(1.74)	0.97(1.74)	0.957	0.1
Baseline number of inpatient visits PPPM	0.19(0.49)	0.2(0.45)	0.193	1.8	0.04(0.19)	0.04(018)	0.007	1.3	0.01(0.05)	0.01(0.05)	0.817	0.4	0(0.03)	0(0.03)	0.654	0.6

Propensity score matching was applied for each cardiovascular disease risk cohort using covariates: age group, gender, US region, baseline Charlson comorbidity index score, Chronic Disease Score, comorbidities (hypertension, arrhythmias, metabolic syndrome, liver disease, obesity and chronic kidney disease) and number of inpatient admissions per patient per month.

*CHD RE* coronary heart disease risk equivalent, *SD* standard deviation, *STD* standardized difference, *CV* cardiovascular, *CVD* cardiovascular disease, *PPPM* per patient per month, *PSM* propensity score matching

^a^Chi-square tests were used to evaluate the statistical significance of differences in categorical variables; student t-tests were used for the continuous variables

### Clinical burden

**Fig. 2:**
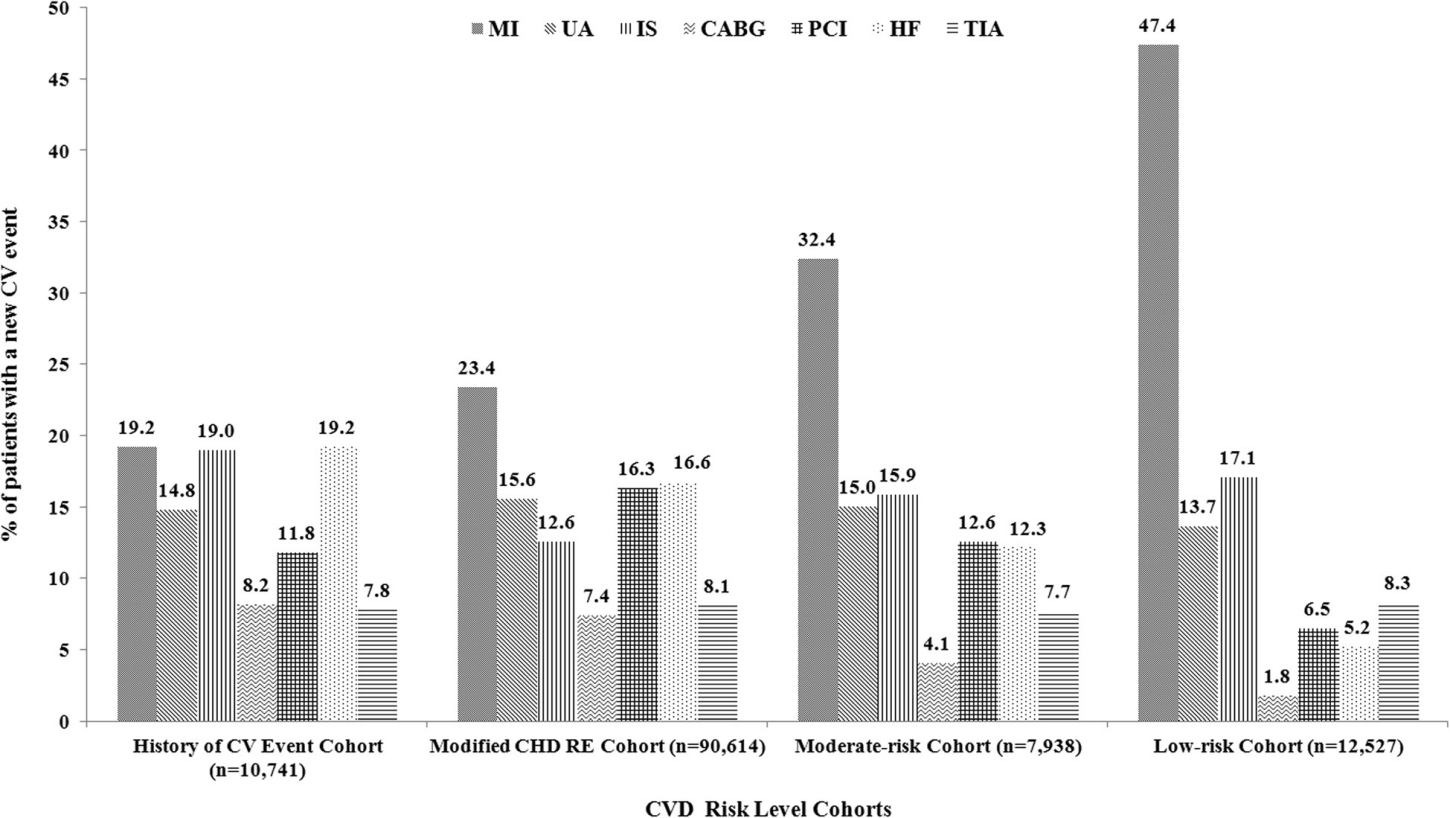
PSM-adjusted Distribution of Index CV Event According to CVD Risk Level. CV: cardiovascular; CVD: cardiovascular disease; PSM: propensity score matching; MI: myocardial infarction; UA: unstable angina; IS: ischemic stroke; CABG: coronary artery bypass graft; PCI: percutaneous coronary intervention; HF: heart failure; TIA: transient ischemic attack; CHD RE: coronary heart disease risk equivalent

MI was more commonly diagnosed than other CV event types among patients in the low-risk, moderate-risk and modified CHD RE cohorts. Frequency of MI, IS and HF was similar among patients in the history of CV event cohort (Fig. 2).

During the 1 month post-index date, among patients with history of a CV event (*n* = 10,741), the mean inpatient length of stay (LOS) was significantly longer among hyperlipidemic patients with a new CV event compared to those without (6.4 vs. 0.25 days, p < 0.0001, Table 2). This trend was observed across all risk cohorts. The inpatient LOS remained longer during the short- and long-term follow-up periods among patients with a new CV event, compared to those without, for all risk cohorts (e.g. history of CV event cohort inpatient LOS in year 2 = 4.14 vs. 1.50 days, p < 0.0001, and in year 3 = 3.72 vs. 1.38 days, p < 0.0001) (Table 3).

**Table 2:** PSM-adjusted follow-up (short and long-term) healthcare utilization for hyperlipidemic patients with and without new CV events, categorized by CVD risk level

	History of CV event cohort			Modified CHD RE cohort			Moderate risk cohort			Low risk cohort		
	Without CV events	With CV events		Without CV events	With CV events		Without CV events	With CV events		Without CV events	With CV events	
	(*N* = 10741)	(*N* = 10741)		(*N* = 90614)	(*N* = 90614)		(*N* = 7938)	(*N* = 7938)		(*N* = 12527)	(*N* = 12527)	
	N/Mean [%]/(SD)	N/Mean [%]/(SD)	P-value^a^	N/Mean [%]/(SD)	N/Mean [%]/(SD)	P-value^a^	N/Mean [%]/(SD)	N/Mean [%]/(SD)	P-value^a^	N/Mean [%]/(SD)	N/Mean [%]/(SD)	P-value^a^
All-cause healthcare utilization 1 month (acute phase) post-index date												
Number of continuous enrollment patients	10577[98.5 %]	10282[95.7 %]	<0.0001	89539[98.8 %]	88196[97.3 %]	<0.0001	7845[98.8 %]	7727[97.3 %]	<0.0001	12428[99.2 %]	12317[98.3 %]	<0.0001
Inpatient LOS (days)	0.25(1.94)	6.43(6.94)	<0.0001	0.07(0.90)	5.22(5.39)	<0.0001	0.04(0.61)	4.97(4.91)	<0.0001	0.01(0.35)	4.42(4.08)	<0.0001
Number of patients with Inpatient Visits	346[3.3 %]	10282[100.0 %]	<0.0001	1102[1.2 %]	88196[100.0 %]	<0.0001	59[0.8 %]	7727[100.0 %]	<0.0001	32[0 .3 %]	12317[100.0 %]	<0.0001
Number of patients with Outpatient ER Visits	387[3.7 %]	1503[14.6 %]	<0.0001	1674[1.9 %]	15425[17.5 %]	<0.0001	124[1.6 %]	1663[21.5 %]	<0.0001	128[1.0 %]	3194[25.9 %]	<0.0001
Number of patients with Outpatient Office Visits	5431[51.3 %]	7141[69.5 %]	<0.0001	39190[43.8 %]	67063[76.0 %]	<0.0001	2857[36.4 %]	5654[73.2 %]	<0.0001	3110[25.0 %]	9311[75.6 %]	<0.0001
Number of patients with Outpatient Visits^b^	7297[69.0 %]	9296[90.4 %]	<0.0001	50185[56.0 %]	81329[92.2 %]	<0.0001	3616[46.1 %]	7078[91.6 %]	<0.0001	3895[31.3 %]	11266[91.5 %]	<0.0001
Number of patients with Outpatient Pharmacy Visits	6842[64.7 %]	7253[70.5 %]	<0.0001	56480[63.1 %]	67216[76.2 %]	<0.0001	4736[60.4 %]	6266[81.1 %]	<0.0001	3500[28.2 %]	9242[75.0 %]	<0.0001
Number of visits (PPPM)												
Inpatient stays	0.04(0.21)	1.18 (0.48)	<0.0001	0.01(0.13)	1.14(0.42)	<0.0001	0.01(0.11)	1.13(0.40)	<0.0001	0.00(0.06)	1.11(0.36)	<0.0001
Outpatient Visits^b^	2.06(2.76)	4.64(4.68)	<0.0001	1.35(2.05)	4.29(4.11)	<0.0001	1.00(1.67)	4.17(4.08)	<0.0001	0.64(1.32)	4.24(4.13)	<0.0001
Outpatient ER Visits	0.04(0.22)	0.17(0.45)	<0.0001	0.02(0.16)	0.20(0.47)	<0.0001	0.02(0.14)	0.25(0.51)	<0.0001	0.01(0.12)	0.29(0.53)	<0.0001
Outpatient Pharmacy Visits	1.76(1.93)	2.29(2.16)	<0.0001	1.43(1.60)	2.34(1.96)	<0.0001	1.21(1.40)	2.28(1.76)	<0.0001	0.47(0.93)	1.74(1.50)	<0.0001
Outpatient Office Visits	1.00(1.50)	1.42(1.49)	<0.0001	0.77(1.31)	1.50(1.41)	<0.0001	0.62(1.19)	1.38(1.35)	<.0001	0.41(0.98)	1.39(1.34)	<0.0001
All-cause Healthcare Utilizations 1 year (31–365 days) post-index date												
Number of continuous enrollment patients	8447[78.6 %]	7808[72.7 %]	<0.0001	75203[83.0 %]	70525[77.8 %]	<0.0001	6588[83.0 %]	6165[77.7 %]	<.0001	10806[86.3 %]	10089[80.5 %]	<0.0001
Inpatient LOS (days)	2.06(13.01)	6.61(20.85)	<0.0001	0.70(4.94)	3.20(13.27)	<0.0001	0.51(3.98)	2.46(11.26)	<.0001	0.23(2.72)	1.75(10.73)	<0.0001
Number of patients with Inpatient Visits	1304[15.4 %]	2916[37.3 %]	<0.0001	6442[8.6 %]	18316[26.0 %]	<0.0001	445[6.8 %]	1439[23.3 %]	<.0001	376[3.5 %]	1747[17.3 %]	<0.0001
Number of patients with Outpatient ER Visits	2062[24.4 %]	2767[35.4 %]	<0.0001	11284[15.0 %]	19597[27.8 %]	<0.0001	876[13.3 %]	1558[25.3 %]	<.0001	1109[10.3 %]	2370[23.5 %]	<0.0001
Number of patients with Outpatient Office Visits	7810[92.5 %]	7423[95.1 %]	<0.0001	70005[93.1 %]	68022[96.5 %]	<0.0001	5692[86.4 %]	5801[94.1 %]	<.0001	8009[74.1 %]	9396[93.1 %]	<0.0001
Number of patients with Outpatient Visits^b^	8216[97.3 %]	7726[98.9 %]	<0.0001	72302[96.1 %]	69833[99.0 %]	<0.0001	6028[91.5 %]	6048[98.1 %]	<.0001	8569[79.3 %]	9777[96.9 %]	<0.0001
Number of patients with Outpatient Pharmacy Visits	6685[79.1 %]	6397[81.9 %]	<0.0001	61668[82.0 %]	59379[84.2 %]	<0.0001	5440[82.6 %]	5411[87.8 %]	<.0001	6611[61.2 %]	8281[82.1 %]	<0.0001
Number of visits PPPM												
Inpatient stays	0.02(0.06)	0.06(0.11)	<0.0001	0.01(0.04)	0.04(0.09)	<0.0001	0.01(0.03)	0.03(0.07)	<.0001	0.00(0.02)	0.02(0.06)	<0.0001
OutpatientVisits^b^	1.81(1.71)	2.62(2.20)	<0.0001	1.28(1.33)	2.13(1.86)	<0.0001	0.95(1.09)	1.74(1.62)	<.0001	0.65(0.88)	1.50(1.47)	<0.0001
Outpatient ER Visits	0.04(0.08)	0.06(0.14)	<0.0001	0.02(0.06)	0.04(0.10)	<0.0001	0.02(0.05)	0.04(0.09)	<.0001	0.01(0.04)	0.03(0.08)	<0.0001
Outpatient Pharmacy Visits	1.66(1.53)	2.05(1.69)	<0.0001	1.37(1.28)	1.87(1.48)	<0.0001	1.18(1.12)	1.80(1.32)	<.0001	0.50(0.75)	1.29(1.11)	<0.0001
Outpatient Office Visits	0.90(0.88)	1.10(0.99)	<0.0001	0.73(0.79)	0.98(0.87)	<0.0001	0.57(0.71)	0.80(0.80)	<0.0001	0.42(0.64)	0.68(0.73)	<0.0001
All-cause healthcare utilization 2 years post-index date												
Number of patients with Inpatient Visits	848[14.3 %]	1660[29.8 %]	<0.0001	5208[8.8 %]	11020[20.7 %]	<0.0001	394[7.7 %]	856[18.4 %]	<0.0001	391[4.4 %]	915[11.7 %]	<0.0001
Number of patients with Outpatient ER Visits	1455[24.5 %]	1845[33.1 %]	<0.0001	9514[16.1 %]	13799[25.9 %]	<0.0001	759[14.9 %]	1134[24.3 %]	<0.0001	963[10.9 %]	1583[20.2 %]	<0.0001
Number of patients with Outpatient Office Visits	5371[90.6 %]	5084[91.2 %]	0.286	54074[91.8 %]	49751[93.5 %]	<0.0001	4433[87.0 %]	4213[90.4 %]	<0.0001	6969[78.8 %]	6944[88.8 %]	<0.0001
Number of patients with Outpatient Visits^b^	5668[95.6 %]	5352[96.0 %]	0.325	55882[94.9 %]	51394[96.6 %]	<0.0001	4669[91.6 %]	4445[95.3 %]	<0.0001	7377[83.4 %]	7286[93.1 %]	<0.0001
Number of patients with Outpatient Pharmacy Visits	4720[79.6 %]	4549[81.6 %]	0.008	48323[82.0 %]	44590[83.8 %]	<0.0001	4228[83.0 %]	4015[86.1 %]	<0.0001	5886[66.6 %]	6324[80.8 %]	<0.0001
All-cause healthcare utilization 3 years post-index date												
Number of patients with Inpatient Visits	563[14.1 %]	961[27.2 %]	<0.0001	3941[8.8 %]	7146[18.6 %]	<0.0001	288[7.4 %]	590[16.9 %]	<0.0001	325[4.5 %]	617[10.3 %]	<0.0001
Number of patients with Outpatient ER Visits	965[24.2 %]	1173[33.2 %]	<0.0001	7049[15.8 %]	9683[25.2 %]	<0.0001	503[12.9 %]	817[23.4 %]	<0.0001	804[11.2 %]	1187[19.9 %]	<0.0001
Number of patients with Outpatient Office Visits	3569[89.4 %]	3150[89.2 %]	0.78	40638[91.0 %]	35419[92.0 %]	<0.0001	3420[87.6 %]	3095[88.8 %]	0.115	5871[81.7 %]	5209[87.2 %]	<0.0001
Number of patients with Outpatient Visits^b^	3775[94.5 %]	3335[94.4 %]	0.819	42139[94.4 %]	36762[95.5 %]	<0.0001	3587[91.9 %]	3272[93.9 %]	0.001	6194[86.2 %]	5468[91.6 %]	<0.0001
Number of patients with Outpatient Pharmacy Visits	3216[80.5 %]	2908[82.3 %]	0.047	36437[81.6 %]	32319[84.0 %]	<0.0001	3221[82.5 %]	2958[84.9 %]	0.006	4991[69.4 %]	4804[80.5 %]	<0.0001

Refer to Table 3 for length of stay and number of visits per patient per month during years 2 and 3 of the follow-up period

*PSM* propensity score matching, *CVD* cardiovascular disease, *CV* cardiovascular, *CHD RE* coronary heart disease risk equivalent, *SD* standard deviation, *LOS* length of stay, *PPPM* per patient per month, *ER* emergency room

^a^Chi-square tests were used to evaluate the statistical significance of differences in categorical variables; student t-tests were used for the continuous variables

^b^Outpatient visits included emergency room, laboratory/pathology, radiology, outpatient surgical or diagnostic procedure and office visits

**Table 3:** PSM-adjusted follow-up (2 years and 3 years) healthcare utilization for hyperlipidemic patients with and without new CV events, categorized by CVD risk level

	History of CV event cohort			Modified CHD RE cohort			Moderate-risk cohort			Low-risk cohort		
	Without CV events	With CV events		Without CV events	With CV events		Without CV events	With CV events		Without CV events	With CV events	
	(*N* = 10,741)	(*N* = 10,741)		(*N* = 90,614)	(*N* = 90,614)		(*N* = 7,938)	(*N* = 7,938)		(*N* = 12,527)	(*N* = 12,527)	
	N/Mean [%]/(SD)	N/Mean [%]/(SD)	P-value^a^	N/Mean [%]/(SD)	N/Mean [%]/(SD)	P-value^a^	N/Mean [%]/(SD)	N/Mean [%]/(SD)	P-value^a^	N/Mean [%]/(SD)	N/Mean [%]/(SD)	P-value^a^
All-cause Healthcare Utilization 2 Years Post-index Date												
Number of Continuous Enrollment Patients	5928[55.2 %]	5576[51.9 %]	<0.0001	58916[65.0 %]	53212[58.7 %]	<0.0001	5096[64.2 %]	4662[58.7 %]	<0.0001	8844[70.6 %]	7822[62.4 %]	<0.0001
Inpatient LOS (days)	1.50(7.21)	4.14(16.22)	<0.0001	0.71(4.81)	2.10(9.46)	<0.0001	0.60(4.09)	1.78(9.78)	<0.0001	0.24(1.70)	0.76(4.36)	<0.0001
Number of Visits (PPPM)												
Inpatient stays	0.02(0.05)	0.04(0.09)	<0.0001	0.01(0.04)	0.03(0.07)	<0.0001	0.01(0.03)	0.02(0.06)	<0.0001	0.00(0.02)	0.01(0.04)	<0.0001
Outpatient Visits^b^	1.72(1.70)	2.07(2.01)	<0.0001	1.26(1.36)	1.63(1.65)	<0.0001	0.97(1.09)	1.27(1.31)	<0.0001	0.73(0.95)	0.97(1.13)	<0.0001
Outpatient ER Visits	0.03(0.08)	0.05(0.13)	<0.0001	0.02(0.06)	0.04(0.09)	<0.0001	0.02(0.05)	0.03(0.08)	<0.0001	0.01(0.04)	0.02(0.07)	<0.0001
Outpatient Pharmacy Visits	1.63(1.52)	1.96(1.70)	<0.0001	1.36(1.28)	1.78(1.49)	<0.0001	1.22(1.15)	1.67(1.34)	<0.0001	0.62(0.87)	1.22(1.14)	<0.0001
Outpatient Office Visits	0.86(0.86)	0.94(0.94)	<0.0001	0.71(0.78)	0.83(0.82)	<0.0001	0.56(0.67)	0.67(0.74)	<0.0001	0.46(0.67)	0.53(0.64)	<0.0001
All-cause Healthcare Utilization 3 Years Post-index Date												
Number of Continuous Enrollment Patients	3994[37.2 %]	3533[32.9 %]	<0.0001	44654[49.3 %]	38489[42.5 %]	<0.0001	3903[49.2 %]	3485[43.9 %]	<0.0001	7188[57.4 %]	5971[47.7 %]	<0.0001
Inpatient LOS (days)	1.38(7.05)	3.72(14.25)	<0.0001	0.69(4.98)	1.77(8.28)	<0.0001	0.72(5.43)	1.68(8.17)	<0.0001	0.29(2.57)	0.72(5.57)	<0.0001
Number of visits (PPPM)												
Inpatient Stays	0.02(0.05)	0.04(0.09)	<0.0001	0.01(0.04)	0.02(0.07)	<0.0001	0.01(0.03)	0.02(0.07)	<0.0001	0.00(0.02)	0.01(0.04)	<0.0001
OutpatientVisits^b^	1.64(1.74)	1.97(2.14)	<0.0001	1.24(1.41)	1.55(1.65)	<0.0001	0.96(1.18)	1.22(1.35)	<0.0001	0.77(0.97)	0.91(1.16)	<0.0001
Outpatient ER Visits	0.03(0.08)	0.05(0.11)	<0.0001	0.02(0.05)	0.03(0.10)	<0.0001	0.01(0.04)	0.03(0.09)	<0.0001	0.01(0.04)	0.02(0.06)	<0.0001
Outpatient Pharmacy Visits	1.62(1.49)	1.94(1.70)	<0.0001	1.36(1.29)	1.75(1.49)	<0.0001	1.22(1.15)	1.60(1.31)	<0.0001	0.69(0.91)	1.20(1.15)	<0.0001
Outpatient Office Visits	0.83(0.86)	0.87(0.89)	0.043	0.70(0.78)	0.79(0.81)	<0.0001	0.55(0.66)	0.65(0.75)	<0.0001	0.48(0.69)	0.51(0.63)	0.012

*PSM* propensity score matching, *CVD* cardiovascular disease, *CV* cardiovascular, *CHD RE* coronary heart disease risk equivalent, *SD* standard deviation, *LOS* length of stay, *PPPM* per patient per month, *ER* emergency room

^a^Chi-square tests were used to evaluate the statistical significance of differences in categorical variables; student t-tests were used for the continuous variables

^b^Outpatient visits included emergency room, laboratory/pathology, radiology, outpatient surgical or diagnostic procedure and office visits

During the 1 month post-index date, patients with history of a CV event (n = 10,741) had significantly more outpatient emergency room (ER) visits PPPM compared to patients without a new CV event (0.17 vs. 0.04 visits, p < 0.0001, Table 2). This trend continued across all risk cohorts and during all follow-up periods (Tables 2 & 3). Among hyperlipidemic patients with new CV events, all resource utilization components were highest during the 1-month post-index follow-up phase for all risk cohorts, indicating that the highest healthcare utilization occurred during the first month post-CV event. However, healthcare resource utilization during years 2 and 3 of the follow-up period remained significantly higher for patients with a new CV event than for those without, across all risk cohorts (e.g. history of CV event cohort ER visits PPPM during year 2 =0.05 vs. 0.03 visits, p < 0.0001; and year 3 = 0.05 vs. 0.03 visits, p < 0.0001).

### Economic burden

Across all CV event type and risk cohorts, the direct incremental costs ranged from $17,903 to $65,825 in the first year of follow-up period, $474 to $19,617 during the second year post-CV event and $2,598 to $26,982 during the third year post-CV event (Table 4).

**Table 4:** Total annual incremental costs for hyperlipidemic patients with new CV events categorized by post-event periods

CV event type	1st year post-CV event				2 Years post-CV event				3 Years post-CV event			
	History of CV event cohort	Modified CHD RE cohort	Moderate-risk cohort	Low-risk cohort	History of CV event cohort	Modified CHD RE cohort	Moderate-risk cohort	Low-risk cohort	History of CV event cohort	Modified CHD RE cohort	Moderate-risk cohort	Low-risk cohort
	Mean [CI]	Mean [CI]	Mean [CI]	Mean [CI]	Mean [CI]	Mean[CI]	Mean [CI]	Mean [CI]	Mean [CI]	Mean [CI]	Mean [CI]	Mean [CI]
Any CV event	$41,168 [$39,130, $43,206]	$41,648 [$41,126, $42,171]	$40,500 [$39,039, $41,960]	$39,869 [$38,768, $40,971]	$9,436 [$7,547, $11,324]	$8,301 [$7,850, $8,753]	$6,622 [$5,267, $7,976]	$5,900 [$5,103, $6,698]	$11,400 [$8,834, $13,966]	$7,386 [$6,834, $7,939]	$6,622 [$5,160, $8,536]	$4,704 [$3,906, $5,502]
MI	$51,686 [$46,728, $56,645]	$52,671 [$51,515, $53,826]	$49,538 [$46,939, $52,137]	$47,840 [$46,131, $49,550]	$10,596 [$4,563, $16,629]	$8,105 [$7,199, $9,010]	$4,935 [$3,249, $6,621]	$5,131 [$4,210, $6,052]	$11,249 [$6,336, $16,162]	$7,052 [$6,059, $8,046]	$5,160 [$2,809, $7,511]	$4,623 [$3,608, $5,639]
IS	$36,572 [$31,751, $41,394]	$36,560 [$34,951, $38,168]	$34,511 [$30,796, $38,227]	$33,791 [$30,996, $36,586]	$7,691 [$3,934, $11,449]	$7,679 [$6,400, $8,958]	$10,009 [$4,623, $15,394]	$5,437 [$3,369, $7,505]	$11,227 [$4,008, $18,446]	$6,652 [$5,200, $8,104]	$4,996 [$799, $9,193]	$4,403 [$1,673, $7,134]
UA	$34,874 [$30,297, $39,451]	$31,627 [$30,649, $32,604]	$31,737 [$28,737, $34,737]	$28,659 [$26,689, $30,629]	$7,108 [$3,350, $10,866]	$6,339 [$5,487, $7,191]	$6,377 [$3,454, $9,299]	$6,015 [$3,666, $8,364]	$7,504 [$1,757, $13,251]	$6,530 [$5,399, $7,660]	$3,626 [$1,375, $5,877]	$3,227 [$1,729, $4,725]
PCI	$32,263 [$28,260, $36,266]	$36,231 [$35,392, $37,070]	$37,246 [$34,028, $40,463]	$38,259 [$35,589, $40,929]	$6,910 [$2,879, $10,941]	$7,583 [$6,734, $8,431]	$7,843 [$4,272, $11,414]	$10,203 [$6,274, $14,131]	$7,972 [$3,150, $12,794]	$6,435 [$5,331, $7,539]	$10,079 [$5,294, $14,864]	$6,579 [$3,227, $9,931]
CABG	$55,548 [$50,438, $60,657]	$65,296 [$63,447, $67,145]	$65,015 [$59,236, $70,794]	$65,825 [$59,970, $71,680]	$583 [−$3,765, $4,930]	$3,380 [$2,269, $4,490]	$474 [−$2,803, $3,751]	$6,414 [$862, $11,966]	$5,081 [−$285, $10,447]	$2,598 [$1,108, $4,088]	$7,902 [−$4,782, $20,586]	$7,716 [$63, $15,369]
HF	$46,890 [$40,421, $53,358]	$45,514 [$43,687, $47,342]	$43,064 [$36,834, $49,293]	$41,001 [$34,370, $47,633]	$19,617 [$13,899, $25,335]	$17,525 [$15,544, $19,507]	$11,289 [$4,682, $17,897]	$11,897 [$5,582, $18,213]	$26,982 [$16,976, $36,989]	$17,638 [$14,810, $20,466]	$17,484 [$8,773, $26,195]	$7,820 [$2,547, $13,093]
TIA	$23,900 [$18,738, $29,062]	$19,055 [$17,835, $20,275]	$17,903 [$14,265, $21,540]	$18,054 [$15,167, $20,940]	$11,557 [$6,392, $16,722]	$5,181 [$3,933, $6,429]	$4,440 [$1,432, $7,447]	$3,941 [$1,177, $6,704]	$11,386 [$1,842, $20,931]	$4,228 [$2,709, $5,748]	$4,405 [−$547, $9,356]	$4,087 [$1,796, $6,378]

*CI* Confidence interval, *CV* cardiovascular, *CHD RE* coronary heart disease risk equivalent, *MI* myocardial infarction, *IS* ischemic stroke, *UA* unstable angina, *PCI* percutaneous coronary intervention, *CABG* coronary artery bypass graft, *HF* heart failure, *TIA* transient ischemic attack

Direct incremental costs categorized by CV event type varied in relation to the duration of the follow-up period. The direct incremental costs accrued during the 1-month post-index phase represented approximately 45-90 % of first-year costs (data not shown). During the first year post-CV event, CABG costs were highest ($55,548–$65,825) for all risk cohorts, followed by MI ($47,840–$51,686) and HF ($41,001–$46,890). During years 2 and 3 post-index date, patients diagnosed with HF incurred the highest cost burden (year 2: $11,289–$19,617; year 3: $7,820–$26,982) among all risk cohorts. The direct incremental costs during these years were mainly driven by heart failure. For all CV event types, first-year incremental costs were higher compared to those accrued in the second and third post-CV event years; second- and third-year costs were always higher for hyperlipidemic patients with new CV events than for their matched patients without CV events.

## Discussion

Our study showed that the long-term clinical and economic burden associated with CV events among hyperlipidemic patients was substantial across all risk cohorts, but especially among high-risk cohorts (i.e. patients with history of a CV event and prior CHD RE diagnosis). Our mean healthcare resource utilization analysis demonstrated that during the acute follow-up period, hyperlipidemic patients with new CV events had an additional +4.4 to +6.2 (days) inpatient LOS and +2.6 to +3.6 outpatient visits PPPM, compared to patients without CV events. The clinical burden remained over the long-term, and was substantial for patients with a new CV event (year 2 incremental inpatient LOS: +0.5 to +2.6, outpatient visits PPPM: +0.2 to +0.3; year 3 incremental inpatient LOS: +0.4 to +2.3, outpatient visits PPPM: +0.1 to +0.3). The pattern of long term healthcare resource utilization among patients with new CV events may be attributable to the higher long term HF costs. Our study also reported that a large proportion (65.8 %) of patients with a new CV event had more than one new CV event during the follow-up period, adding to the long-term clinical burden of CV events on hyperlipidemic patients. These are only the direct medical costs of care; total costs would be larger if other indirect costs associated with CVE were accounted for. A prior study did show that new CVE were associated with increased indirect costs [30].

Previous studies have reported that inpatient hospital stays and ER visits are expensive, resource intensive and impose a great clinical burden on patients [31, 32]. Higher healthcare resource utilization is a major component of increased healthcare costs. Healthcare costs were higher among hyperlipidemic patients with a new CV event in the acute phase, compared to patients without a new CV event. Our results are similar to the Chapman et al. study, which concluded that patients with new CV events incurred the highest follow-up costs during the acute phase, and acute phase costs were much higher than those in years 2 and 3 [7]. However, our study also determined that incremental costs remained higher through 3 years of follow-up (year 1: $39,869 to $41,648 higher; year 2: $5,900 to $ 9,436 higher; year 3: $4,704 to $11,400 higher), for all risk cohorts of hyperlipidemic patients with a new CV event, compared to those without, emphasizing a sustained economic burden. Compared with the Chapman et al. [7] study with cost estimates from 2001–2006, the present study also provides more recent estimates for healthcare resource utilization and costs across the CVD risk spectrum (history of CV events through low risk) rather than excluding the highest risk cohort (i.e. patients with a history of CV events) as in the Chapman et al. study. Our study also captures the cost of care for multiple CV events thereby providing a more accurate estimate of the direct cost of care for patients experiencing new CV events rather than estimating the cost for each specific CV event type. Setting potentially arbitrary time cut-points to distinguish between different CV events among patients with multiple events may produce artificial cost results, as some CV events may occur with little time gap and the cost of one event is entwined with the cost of the next event.

Our study also brings to light the noteworthy clinical and economic burden among patients in the high-risk cohorts (i.e., history of CV event and modified CHD RE cohorts). Inpatient LOS was, on average, 0.09 to 4.89 days longer among patients with history of a CV event or modified CHD RE, compared to those at moderate or lower risk, during all follow-up time periods, suggesting that high-risk patients have greater healthcare resource needs. During the long-term post-CV event periods (1, 2 and 3 years follow-up), patients with a new CV event in the higher risk cohorts utilized more incremental ER visits PPPM, compared to those in the moderate- and low-risk cohorts, demonstrating the potential for a higher healthcare cost burden during the longer-term post-CV event periods. Future research is warranted to more specifically determine the underlying reasons for the sustained difference in clinical and economic burden between high-risk hyperlipidemic patients with a CV event and those without a CV event.

Our study results were similar to a study done by Karan et al., indicating similarity in findings that CVD had more outpatient and inpatients stays and economic burden of CVD is large [33]. However, this study utilized a national survey of households in India and focused on out-of-pocket spending and non-medical spending for CVD, whereas our present study focused on a patient-level perspective of direct medical costs for new CV events. Although previous studies provide a general frame of reference, the cost estimates are not directly comparable to the incremental direct costs in the present study since the studies differed in study design (matched cohorts versus survey sample) [34] and composition of the study population (US hyperlipidemia patients versus hypertension or solely acute coronary syndrome patients including those residing in developing countries) [33, 34], sample size (*n* = 10,741 vs. 4,669) [10], CVD risk level (low through high CV risk vs. exclusion of high secondary prevention patients) [7, 8] and contemporaneous cost estimates (2009–2013 vs. 2001–2006) [7]. Due to considerable variation in costs by CV event type, the results of our analysis strengthen the importance of evaluating total and individual CV event costs, as this specific information may be essential for secondary prevention and treatment decisions for high-risk patients.

Our present study demonstrated the sustained high economic and clinical burden associated with the occurrence of CV events among hyperlipidemic patients. In patients who have already experienced or who are at high risk for experiencing a CV event, lifestyle intervention strategies alone may not be sufficient to maximally reduce CVD risk [35, 36]. Current US treatment guidelines recommend lipid-lowering therapy in addition to lifestyle modifications to lower LDL-C levels for primary and secondary prevention for high-risk individuals [37]. Although statins are widely prescribed for elevated LDL-C levels, 9 %–20 % of treated patients, especially high-risk patients, continue to have elevated LDL-C and remain at risk for new CV events [38]. Potential new pharmacological treatments (e.g. anti-proprotein convertase subtilisin/kexin type 9 [PCSK9] monoclonal antibodies) aimed at significantly lowering LDL-C beyond that of current available treatment options [39], could potentially help to reduce the substantial clinical and economic burden.

### Limitations

Our study limitations were primarily related to the retrospective use of claims data [7, 15]. Misclassification of CV risk, although it cannot be quantified, is likely to be low since the ICD-9-CM codes utilized to capture history of CVD included codes for old MI, stroke sequelae, etc. that would include a history of CVD beyond the baseline period. Similarly, important patient information, including blood pressure, smoking history and family history was not available in the claims data to more accurately classify patients within the CHD RE cohort. Also, administrative claims data do not offer information on whether an elective procedure (CABG, PCI) was planned, thus planned procedures could not be completely excluded from the study. Nevertheless, utilization of the PSM method reduced the differences between patients with and without new CV events and created a balanced study cohort, such that healthcare utilization and incremental costs were more accurately compared.

## Conclusion

Substantial incremental costs and healthcare resource utilization 1 month up to 3 years post-CV event highlight the short- and long-term economic and clinical burden especially on high-risk hyperlipidemic patients and the US healthcare system. Interventions used to prevent or reduce the occurrence of CV events among patients with hyperlipidemia may result in substantial cost savings and reduce the clinical burden in the United States.

## Appendix 1

**Table 5:** Cardiovascular event identification codes

Cardiovascular events	Diagnosis/Procedure codes
Myocardial Infarction	ICD-9-CM: 410.xx
Unstable Angina	ICD-9-CM: 411.1x, 411.8x
Ischemic Stroke	ICD-9-CM: 433.x1, 434.x1
Coronary Artery Bypass Graft	CPT: 33510-33514, 33516-33519, 33521-33523, 33530, 33533-33536
	HCPCS: S2205-S2209
	ICD-9-CM: 36.10-36.17, 36.19
Percutaneous Coronary Intervention	ICD-9-CM: 00.66, 36.06, 36.07, 17.55
	CPT: 92980, 92981, 92982, 92984-92996, 92973
	HCPCS: G0290, G0291
Transient Ischemic Attack	ICD-9-CM: 435.0x, 435.1x, 435.8x, 435.9x
Heart Failure	ICD-9-CM: 428.xx

*CPT* Current Procedural Terminology, *HCPCS* Healthcare Common Procedural Coding System, *ICD-9-CM* International Classification of Diseases, Ninth Revision, Clinical Modification

## Appendix 2

**Table 6:** Cardiovascular risk levels and codes, modified based on NCEP ATP III guidelines

Risk Level		Code
History of CV event	Myocardial infarction	ICD-9-CM: 410, 412
	Unstable angina	ICD-9-CM: 411.1, 411.81, 411.89
	Coronary artery bypass graft	CPT-4: 33503-33545
	Percutaneous coronary intervention	ICD-9 Procedure: 00.66, 36.09
	Ischemic Stroke	ICD-9 CM: 434, 436, 437.0, 437.1, 438, 997.02
Modified CHD RE	Peripheral arterial disease	ICD-9-CM: 440.0x-440.4x, 440.8x-440.9x, 443.81, 443.9x
	Abdominal aortic aneurysm	ICD-9-CM: 441.3x-441.4x
	Coronary artery disease	ICD-9-CM: 433.1x
	Diabetes	ICD-9-CM: 249.xx-250.xx
	Dyslipidemia	ICD-9-CM: 272.0x-272.4x
Moderate risk	At least two of the following three risk factors identifiable from administrative claims data: a) hypertension (ICD-9-CM code or pharmacy claim for a blood pressure–lowering agent), b) age 45 years or older for men and 55 years or older for women c) pre-index high-density lipoprotein (HDL) cholesterol below 40 mg/dl.	Hypertension: ICD-9-CM codes 401.1-401.9, 642.00-642.04, 401.0, 437.2, 402.00-405.99, 642.10-642.24, 642.70-642.94
Low risk	Zero or one risk factor	

*CHD RE* coronary heart disease risk equivalent, *CV* cardiovascular, *ICD-9-CM* International Classifications of Diseases, 9^th^ Revision Clinical Modifications, *CPT* Current Procedural Terminology, *NCEP* *ATP III* National Cholesterol Education Program Adult Treatment Panel III

## Appendix 3

**Table 7:** 12-month Pre-index demographic and clinical characteristics for hyperlipidemic patients with and without new CV events before matching

	History of CV event cohort				Modified CHD RE cohort				Moderate risk cohort				Low risk cohort			
	Without CV events	With CV events			Without CV events	With CV events			Without CV events	With CV events			Without CV events	With CV events		
	(*N* = 10744)	(*N* = 77163)			(*N* = 145642)	(*N* = 156793)			(*N* = 11816	(*N* = 14544)			(*N* = 16083)	(*N* = 18665)		
	Mean [%]/(SD)	Mean [%]/(SD)	P-value^a^	STD	Mean [%]/(SD)	Mean [%]/(SD)	P-value^a^	STD	Mean [%]/(SD)	Mean [%]/(SD)	P-value^a^	STD	Mean [%]/(SD)	Mean [%]/(SD)	P-value^a^	STD
Age	73.66 (13.15)	66.41 (13.65)	<0.0001		65.28 (13.16)	65.17 (13.17)	0.0189		67.83 (12.63)	65.83 (12.83)	<0.0001		57.73 (12.45)	54.56 (11.01)	<0.0001	
18-24	[0.0 %]	[0.0 %]	0.04	2.8	[0.03 %]	[0.05 %]	0.0018	1.1	[0.00 %]	[0.00 %]	N/A	0.0	[0.2 %]	[0.2 %]	0.3062	1.1
25-34	[0.1 %]	[0.4 %]	<0.0001	6.6	[0.3 %]	[0.4 %]	0.0002	1.4	[0.00 %]	[0.00 %]	N/A	0.0	[1.3 %]	[1.8 %]	0.0016	3.4
35-54	[6.9 %]	[18.4 %]	<0.0001	34.8	[19.6 %]	[19.6 %]	0.6784	0.2	[11.7 %]	[16.7 %]	<0.0001	14.3	[41.3 %]	[51.1 %]	<0.0001	19.8
55-64	[22.1 %]	[32.1 %]	<0.0001	22.7	[35.7 %]	[35.3 %]	0.0122	0.9	[38.0 %]	[41.2 %]	<0.0001	6.6	[34.7 %]	[33.6 %]	0.0327	2.3
≥65	[70.9 %]	[49.2 %]	<0.0001	45.6	[44.4 %]	[44.7 %]	0.1341	0.5	[50.3 %]	[42.1 %]	<0.0001	16.5	[22.5 %]	[13.3 %]	<0.0001	24.1
Male	[66.6 %]	[63.0 %]	<00001	7.7	[62.2 %]	[61.1 %]	<0.0001	2.4	[62.4 %]	[65.2 %]	<0.0001	5.9	[60.2 %]	[66.4 %]	<0.0001	0.1
US geographic region																
Northeast	[39.3 %]	[36.0 %]	<0.0001	6.8	[35.3 %]	[34.9 %]	0.0398	0.7	[33.1 %]	[31.0 %]	0.0003	4.5	[35.8 %]	[31.4 %]	<0.0001	9.4
Midwest	[22.1 %]	[25.5 %]	<0.0001	8.0	[26.2 %]	[26.2 %]	0.2635	0.4	[27.2 %]	[28.2 %]	0.0671	2.3	[25.0 %]	[28.4 %]	<0.0001	7.6
South	[24.4 %]	[26.3 %]	<0.0001	4.4	[26.8 %]	[27.3 %]	0.0006	1.3	[26.0 %]	[28.3 %]	<0.0001	5.2	[27.8 %]	[30.4 %]	<0.0001	5.7
West	[14.2 %]	[12.2 %]	<0.0001	5.9	[11.5 %]	[11.5 %]	0.864	0.1	[13.7 %]	[12.5 %]	0.004	3.6	[11.3 %]	[9.8 %]	<0.0001	5.0
Baseline comorbid condition																
Charlson comorbidity index (CCI)	2.72(2.15)	3.30(2.65)	<0.0001	24.3	0.99(1.49)	2.10(2.27)	<0.0001	57.6	0.53 (1.23)	0.92(1.69)	<0.0001	26.6	0.22(0.78)	0.33 (1.05)	<0.0001	11.4
Chronic disease score	5.28(4.06)	6.01(4.44)	<0.0001	17.0	3.91(3.48)	5.70(4.24)	<0.0001	46.2	4.17 (3.04)	4.70(3.40)	<0.0001	16.6	1.16(1.94)	1.28 (2.22)	<0.0001	5.8
Baseline number of inpatient visits PPPM	0.19(0.49)	0.39(0.84)	<0.0001	28.8	0.04(0.19)	0.18(0.52)	<0.0001	36.1	0.03 (0.19)	0.10(0.35)	<0.0001	24.3	0.01 (0.07)	0.03 (0.20)	<0.0001	15.6

*CHD RE* coronary heart disease risk equivalent, *SD* standard deviation, *STD* standardized difference, *CV* cardiovascular, *CVD* cardiovascular disease, *PPPM* per patient per month

^a^Chi-square tests were used to evaluate the statistical significance of differences in categorical variables; student t-tests were used for the continuous variables
